# An evaluation of potential reference genes for stability of expression in two salmonid cell lines after infection with either *Piscirickettsia salmonis *or IPNV

**DOI:** 10.1186/1756-0500-3-101

**Published:** 2010-04-14

**Authors:** Andrea A Peña, Niels C Bols, Sergio H Marshall

**Affiliations:** 1 Laboratorio de Genética e Inmunología Molecular, Instituto de Biología, Pontificia Universidad Católica de Valparaíso, Av. Brasil 2950, Valparaíso, Chile; 2 Department of Biology, University of Waterloo, 200 University Avenue West, Waterloo, ON N2L 3G1, Canada

## Abstract

**Background:**

Due to the limited number of species specific antibodies against fish proteins, differential gene expression analyses are vital for the study of host immune responses. Quantitative real-time reverse transcription PCR (qRT-PCR) is one of the most powerful tools for this purpose. Nevertheless, the accuracy of the method will depend on the careful selection of genes whose expression are stable and can be used as internal controls for a particular experimental setting.

**Findings:**

The expression stability of five commonly used housekeeping genes [beta-actin (*ACTB*), elongation factor 1-alpha (*EF1A*), ubiquitin (*UBQ*), glyceraldehyd-3-phosphate dehydrogenase (*GAPDH*) and tubulin alpha (*TUBA*)] were monitored in salmonid cell lines CHSE-214 and RTS11 after infection with two of the most fastidious fish pathogens, the facultative bacterium *Piscirickettsia salmonis *and the aquabirnavirus IPNV (Infectious Pancreatic Necrosis Virus). After geNorm analysis, *UBQ *and *EF1A *appeared as the most stable, although *EF1A *was slightly upregulated at late stages of *P. salmonis *infection in RTS11. *ACTB *instead, showed a good performance in each case, being always considered within the three most stable genes of the panel. In contrast, infection-dependent differential regulation of *GAPDH *and *TUBA *was also demonstrated.

**Conclusion:**

Based on the data presented here with the cell culture models CHSE-214 and RTS11, we suggest the initial choice of *UBQ*, *ACTB *and *EF1A *as reference genes in qRT-PCR assays for studying the effect of *P. salmonis *and IPNV on the host immune response.

## Background

To date, cDNA microarray and quantitative real-time reverse transcription PCR (qRT-PCR) have become the most important and reliable tools to study differential gene expression in fish, where species-specific antibodies are scarce. Although qRT-PCR combines advantages of specificity, sensitivity, speed, throughput and reproducibility over conventional methods an accurate normalization of data is fully required [1]. Errors in the quantification of mRNA transcripts arise from any variation in the amount of starting material between samples. A common strategy to overcome this problem is to simultaneously amplify a non-regulated housekeeping gene with those targeted to allow quantitative normalization of the experimental cDNA inputs. However, it has also been demonstrated that expression levels of these genes may vary considerably depending on cell types, tissues, experimental treatments and even under different diseases [2]. Moreover, the use of a single reference gene for normalization is nowadays discouraged by an increasing number of authors [3-5]. Consequently, it is highly necessary to validate their constitutive expression for a particular experimental setting and therefore a crucial component when assessing a new model [6].

The present study aims to validate the usefulness of five potential housekeeping genes for normalization of a number of salmonid relevant immune genes. We are currently developing SYBR Green based real-time assays for studying the host immune response influenced by the infection with the facultative bacterium *Piscirickettsia salmonis *and with the IPNV, respectively. The *in vitro *models CHSE-214 (an epithelial-like embryo cell line derived from Chinook salmon, *Oncorhynchus tshawitscha*) and RTS11 (monocyte/macrophage-like cell line derived from rainbow trout, *Oncorhynchus mykiss*) have been of great help for this purpose because they have been shown to be susceptible to a wide range of viral infections [7,8] and to *P. salmonis *[9,10]. The potential reference genes we have examined are beta-actin (*ACTB*), elongation factor 1-alpha (*EF1A*) and glyceraldehyd-3-phosphate dehydrogenase (*GAPDH*), which have been previously validated in several studies on diverse fish species including salmonids [11-13], and ubiquitin (*UBQ*) and tubulin alpha (*TUBA*), which have been reported for fish species like the three-spine-stickelback (*Gasterosteus aculeatus *[14]) and zebrafish (*Danio rerio *[15]), but not in salmonids. The five housekeeping genes were selected based on their previous use as internal controls for gene expression studies, the availability of housekeeping gene sequences for salmonids and related teleost species, and because they have roles in different cellular functions (Table 1), thus reducing the likelihood that they exhibited regulated covariation.

**Table 1:** Name and function of candidate reference genes

Symbol	Gene name	Function	GenBank accession no. (*Oncorhynchus mykiss*)
*ACTB*	Beta actin	Cytoskeletal structural protein	emb|AJ438158.1|OMY438158
*EF1A*	Elongation factor 1 alpha	Protein biosynthesis	gb|AF498320.1|
*GAPDH*	Glyceraldehyd-3-phosphate-dehydrogenase	Glycolytic enzyme	gb|AF027130.1|AF027130
*UBQ*	Ubiquitin	Protein degradation	dbj|AB036060.1|
*TUBA*	Tubulin alpha	Structural protein	NM_001124691.1

## Methods

CHSE-214 was obtained from the American Type Culture Collection (ATCC CRL-1681), whereas RTS11 was developed by the middle author [16] (University of Waterloo, Canada). The routine growth of these cell lines has been described previously [17,18]. Briefly, CHSE-214 cultures were maintained at 17°C in MEM (Gibco) supplemented with 10 mM NaHCO_3_, 15 mM HEPES and 5% FBS (Gibco). RTS11 cultures were maintained at 20°C in Leibovitz's L-15 medium (Gibco) supplemented with 5% FBS. The cultures were free of mycoplasma, as determined by qualitative PCR.

*Piscirickettsia salmonis *type strain LF89 ATCC VR 1361 was grown in the cell line CHSE-214 as described previously [19]. Bacteria obtained from the culture supernatant of 15 days post-infection CHSE-214 cells were used to inoculate CHSE-214 and RTS11 cultures in 25 cm^2 ^plastic tissue culture flasks (Orange) at a concentration of 4.0 × 10^5 ^cells/ml. Prior to inoculation, 1 ml aliquots from infected CHSE-214 culture were centrifuged for 10 min at 900 × g at 4°C to remove debris. The supernatants were transferred to fresh tubes and centrifuged for 30 min at maximal speed at 4°C to concentrate the bacteria. After the supernatants had been discarded, bacteria pellets were resuspended in the medium appropriate for each cell line. The titre of *P. salmonis *used in inoculums was 1 × 10^6.8 ^ml^-1^. This titre was determined on CHSE-214 cells and calculated by the method of Reed and Muench [20]. For expression studies, cells were harvested at 2, 5 and 9 days post-infection.

Experiments with IPNV were performed using the Chilean strain VR299. The virus was propagated by inoculating CHSE-214 cell monolayers at a multiplicity of infection (MOI) of 0.1 to 1 PFU/cell in MEM supplemented with 2% FBS and antibiotics. Infected cultures were incubated at 17°C and monitored until CPE was evident and the clarified supernatants were divided into aliquots that were stored at -20°C. Aliquots were titrated in a plaque formation assay as described previously [21]. For expression studies, both CHSE-214 and RTS11 cultures were inoculated at a MOI of 1×, and cells were harvested at 6 h, 24 h and 48 h post-infection.

All experiments were carried out using three biological replicates, i.e. three independent tissue culture bottles for each time point and assayed independently. Controls were done alike. Times for harvesting *P. salmonis*- and IPNV- infected cultures were chosen as CPE advanced, but making sure that no significant cell death and lysis were taking place.

Total RNA extraction from cell cultures was carried out using Trizol^® ^(Invitrogen) according to the manufacturer instructions. A NanoDrop ND 1000 spectrophotometer was employed to analyze RNA concentration and purity. All samples were DNase treated (RQ1 RNase-free DNase, Promega) to remove any contaminating DNA. For PCR amplification, first strand cDNA was synthesized from 1 μg total RNA using oligo(dT) primer and the AffinityScript™ QPCR cDNA Synthesis Kit (Stratagene).

CHSE-214 and RTS11 cultures were monitored for infection by phase contrast microscopy (Olympus IMT-2 microscope). *P. salmonis *PCR confirmation was carried out by using the primer pair RTS1/RTS4 against the ITS region of the bacterial 16S rRNA operon as described previously [22]. IPNV infections were confirmed by using 1 step RT-PCR procedure (Brilliant QRT-PCR Master Mix Kit 1-Step, Stratagene) with primer set VP2SNP-F/VP2SNP-R (Santi, unpublished) against to the *VP2 *fragment sequence. Reverse transcription was performed by incubating at 50°C for 55 min followed by PCR amplification (95°C for 10 min, 35 cycles of 30 s at 95°C, 30 s at 55°C and 30 s at 72°C, and 72°C for 10 min). Mycoplasma contamination was absent as tested by amplifying with the primer set MyF1/MyR1 (PCR Mycoplasma Detection Set - TaKaRa Biomedicals Takara Shuzo).

Five reference genes (*ACTB*, *UBQ*, *EF1A*, *GAPDH *and *TUBA*), belonging to different functional classes, were selected to reduce the chance of their co-regulation (Table 1). All primers were designed on conserved regions so that they could amplify each gene in both species under study: *O. mykiss *and *O. tschawitcha*. Primers were evaluated with the OligoCalc application [23] to check annealing temperatures and self-complementarity. The specificity of the primers was tested using BLAST analysis against the nr NCBI database. Primer specifications are summarised on Additional file 1. The desired amplicon length (182 - 204 base pairs) was chosen to be similar among all genes to avoid significant differences in PCR efficiencies due to amplicon length. PCR products were cloned into TOPO vector (pCR 2.1, Invitrogen) and submitted to sequencing for verification. Partial *O. tschawitcha *sequences were deposited into GenBank accession numbers: FJ890356 (*EF1A*), FJ890357 (*ACTB*), FJ890359 (*UBQ*) and FJ890358 (*TUBA*).

qRT-PCR was carried out using a MJ Research real-time cycler. Each reaction for amplification of housekeeping candidates contained: 10 μl of the Brilliant II SYBR Green qPCR Master Mix (Stratagene), 100 nM of forward and reverse primers and 2 μl of 10-fold diluted cDNA, to a final volume of 20 μl. PCR was achieved with 10 min activation and denaturation step at 95°C, followed by 40 cycles of 30 s at 95°C, 30 s at the specific annealing temperature (see Additional file 1), 30 s at 72°C and 2 s at 74°C for fluorescence measurement. Following the final cycle, melting curve analysis were performed to examine the specificity in each reaction tube (absence of primer dimers and other non-specific products) by heating the samples from 60 to 90°C in 0.2°C increments with a dwell time at each temperature of 5 s while continuously monitoring the fluorescence.

PCR efficiencies were calculated for each tissue culture cell line using a relative standard curve derived from a pooled cDNA mixture (a ten-fold dilution series with five measuring points). The pooled cDNA was obtained from control and infected samples from both CHSE-214 and RTS11 cell cultures, respectively, using the same RNA isolation and cDNA synthesis protocols as described above. The real-time PCR efficiencies were calculated from the slope according to the established equation E = 10 ^(-1/slope) ^[4].

Real-time PCR were assayed on every biological replicate and each sample was run in duplicate. Each PCR reaction included reverse transcriptase negative controls for testing genomic DNA contamination and a non template negative control to check for primer dimer. To minimize experimental variation, each gene was quantified on the same batch of cDNA and the same gene was tested on the different samples in the same PCR run.

The threshold cycle (Ct) values of the Opticon Monitor 2 software version 2.03 were transformed to relative quantities for analysis with the geNorm 3.5 software as described by Vandesompele et al. [3]. For the conversion of the Ct values to relative quantities [24], reaction efficiencies were used. Relative gene expression for *GAPDH*, *TUBA *and *EF1A *were calculated using the geometric mean of the three most stable genes of each assay as normalization factors and the 2d (in *P. salmonis *assays) and the 6 h (in IPNV assays) controls as calibrators, respectively.

A Mann-Whitney test was used to determine significant differences in gene expression between groups and the calibrator samples. Significance was set at P < 0.05. These last statistical analyses were done using the SPSS 13.0 statistic package.

## Results and Discussion

**Figure 1:**
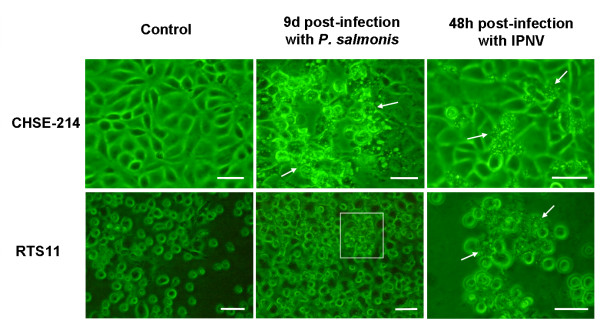
**Microscopic evidence of *Piscirickettsia salmonis *and IPNV infections**. Phase contrast microscope appearance of cultures of CHSE-214 (top row) and RTS11 (bottom row) after the addition of either *Piscirickettsia salmonis *(middle column) or IPNV (right column). The cytopathic effect is seen as morphological changes (arrows) and as homotypic aggregation [highlighted by a box] in the case of RTS11 and *P. salmonis*. In CHSE-214 cultures, *P. salmonis *infection displayed the characteristic vacuolisation of cells. This CPE was visible in small groups of cells as early as three days after infection. Five days post-infection, vacuolisation was extended to approximately 40% of the population and nine days later around 80% displayed CPE. At this point some cells were detaching from the monolayer. In contrast to CHSE-214, RTS11 showed no evidence of vacuolization after infection, but homotypic aggregation of these monocyte-like cells became perceptible as infection advanced. Significant cell death and lysis took place at least 12 days post-infection. IPNV infection was evidenced by rounding up and blebbing of the plasma membrane in CHSE-214 cultures. As infection advanced, apoptotic bodies could be seen. Two days post-infection, over 50% of the population displayed CPE. For RTS11 cultures, strong homotypic aggregation became apparent 24 h post-infection followed by significant morphological changes such as plasma blebbing.

**Figure 2:**
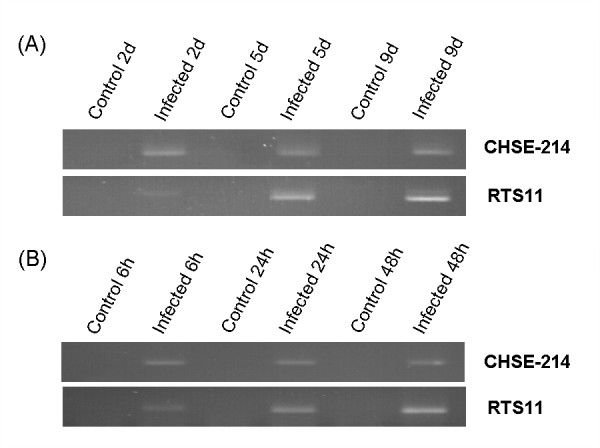
**Evidence of infection by PCR**. PCR confirmation that cultures of CHSE-214 and RTS11 had been infected with either *P. salmonis *(A) or IPNV (B). *P. salmonis *was revealed by primer specific amplification of the ITS region of the bacteria 16S operon (A) and IPNV by primer specific amplification of the *VP2 *fragment (B). These results rule out persistency of infection in control samples, as they showed no primer specific amplification.

Intracellular pathogens such as bacteria and viruses modulate key cellular processes which may involve changes in reference gene expression [25,26]. Each pathogen manipulates several cellular transcription pathways in different degrees also according to the affected cell type [25,26]. Efficient infections with *P. salmonis *and IPNV have been evidenced by increasing cytopathic effect, as well as by accumulation of bacterial DNA and viral RNA over time in both CHSE-214 and RTS11 cell cultures (Figures 1 and 2). To rule out persistency, we made sure CPE was detectable resulting in productive infection (Figure 1). As well, no primer specific amplification of the ITS region of the bacteria 16S operon and the *VP2 *fragment of IPNV were evidenced in control samples (Figure 2). Despite progressive pathogen replication and accumulation in cultures, the expression of some reference genes remained constant, while others varied.

**Figure 3:**
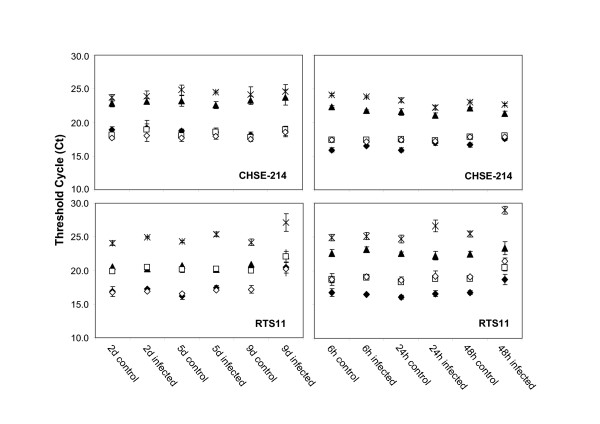
**Transcriptional levels of candidate housekeeping genes measured by QPCR**. Transcriptional levels (Ct values) of potential reference genes (◆ *ACTB*, □ *EF1A*, ▲ *G3PDH*, ○ *UBQ*, ж *TUBA*) in cultures of either CHSE-214 (top panels) or RTS11 (bottom panels) at times after the addition of either *P. salmonis *(left panels) or IPNV (right panels). Each mark shows the mean Ct value (average of three biological replicates) included standard deviations.

Prior validation of the selected reference gene candidates (*ACTB*, *EF1A*, *GAPDH*, *UBQ *and *TUBA*), general expression levels based on mean qPCR threshold cycle (Ct) values in control CHSE-214 and RTS11 cells were determined, since extremely high or low expression levels might preclude their usefulness as internal controls (Figure 3). Ct values from 16.17 and 24.99 indicated expression levels in an appropriate range.

For each assay, a standard curve for RTS11 and for CHSE-214 samples was generated by using 10-fold serial dilutions of pooled cDNA, generated from a mix of infected and control samples. Linear correlation coefficient (R^2^) varying from 0.9910 to 0.9998 and efficiencies between 87% and 96% showed that these assays were suitable for quantitative purposes (see Additional file 1).

**Figure 4:**
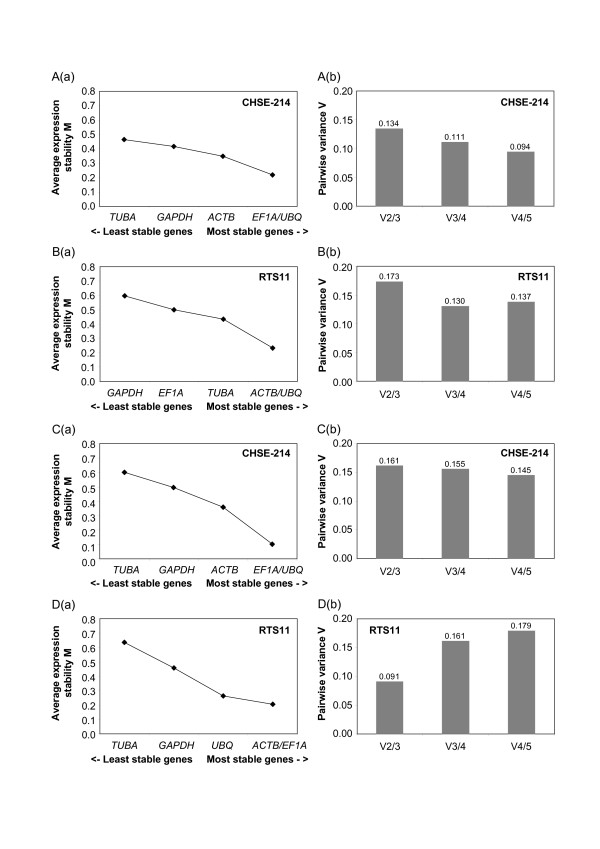
**Programmed analysis of expression stability of the five putative reference genes**. (a) Average expression stability values (M) of the potential reference genes after stepwise exclusion of the least stable gene calculated by the statistical software geNorm, plotted from least stable (left) to most stable (right). (b) Pairwise variation analysis between the normalization factors NF_n _and NF_n+1_, to determine the optimal number of control genes for normalisation. A and B, *P. salmonis *infection experiment, C and D, IPNV infection experiment.

The stability of gene expression over different samples can be achieved by evaluating qRT-PCR data with statistical algorithms. The geNorm software was chosen, since several studies have not found large differences between this tool and the NormFinder and the Bestkeeper [14,26,27]. GeNorm permitted to determine the internal control gene-stability measure M, which was calculated from the average pairwise variation of a particular gene with all other control genes. Genes with the lowest M values have the most stable expression. Assuming that the control genes are not co-regulated, stepwise exclusion of the gene with the highest M value results in a combination of two constitutively expressed housekeeping genes that have the most stable expression in the tested samples [3]. The ranking of the 5 candidate reference genes according to their M value was very similar in all assays (Figure 4a).

In order to determine the optimal number of reference genes required for accurate normalisation, the pairwise variations V_n/n+1 _between each combination of sequential normalisation factors NF_n _containing an increasing number of genes were calculated (Figure 4b). If the addition of a gene produced large differences between consecutives V_n/n+1_, then the added gene should be preferably included for calculation of NF_n _[3]. Following this criteria, only the IPNV infection assay on RTS11 cell cultures might consider the inclusion of a 4^th ^gene. For all other experimental settings, the optimal reference genes should be three.

**Figure 5:**
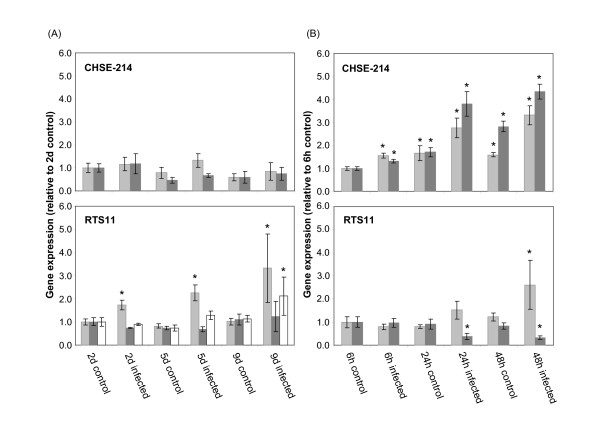
**Normalized *GAPDH*, *TUBA *and *EF1A *gene expression**. Relative mRNA levels for *GAPDH *(grey bars), *TUBA *(dark grey bars) and *EF1A *(white bars) at times after the addition of either *P. salmonis *(A) or IPNV (B). Gene expression was normalized by the geometric mean of the three most stable housekeeping genes determined by geNorm in each assay. The 2d and the 6 h controls were used as calibrators (value = 1) in the (A) *P. salmonis *and in the (B) IPNV assays, respectively. Bars represent the mean values (± SE) for samples from three biological replicates. The asterisk (*) denotes significant differences from calibrators (P < 0.05; Mann-Whitney test).

*UBQ *and *EF1A *were the best ranked reference gene candidates, as they had the lowest sum_p _values (0.813 and 1.023, respectively) which represents the standard deviation (SD) of reference gene expression over all infections investigated (Table 2). *EF1A *has been demonstrated to be a good housekeeping gene for the analysis of infection challenges, although a slight upregulation in RTS11 cells after 9 days *P. salmonis *infection (Figure 5A). In contrast to the reports of modulated expression by viral infections of human cell lines [26] and in infection of Atlantic salmon with ISAV [11], *ACTB *showed in general a good performance during *P. salmonis *and IPNV infection, being considered in all cases as one of the three most stable genes (Figure 4a). As well, its reliability has been confirmed in LPS stimulated cells and tissues of fish [12] and birds [27].

**Table 2:** Results of geNorm analyses

	*GAPDH*	*ACTB*	*EF1A*	*UBQ*	*TUBA*
**CHSE-214-Ps**	0.418	0.350	0.217	0.217	0.465
**RTS11-Ps**	0.596	0.232	0.500	0.232	0.432
**CHSE214-IPNV**	0.496	0.358	0.101	0.101	0.599
**RTS11-IPNV**	0.457	0.205	0.205	0.263	0.636

**Sum_p_**	1.967	1.145	1.023	0.813	2.132

The standard deviations of reference gene expression as determined by geNorm are shown.

(Sum_p_: sum of pathogen infection geNorm values)

In order to show how transcription profile of the least stable genes can be affected during each experimental setting, their relative transcription levels were determined using the normalization factor calculated from the geometric mean of the three most stable housekeeping genes (Figure 5). In agreement with several other studies on teleosts, *GAPDH *was one of the least stable genes in our panel [13,15,28]. In mammals *GAPDH *was shown to be influenced by a large number of physiological states and to play a role in a broad range of cellular mechanisms [29]. *GAPDH *expression was increasingly upregulated in RTS11 cells infected with *P. salmonis *and was slightly upregulated over 48 h in both control and IPNV-treated CHSE-214 cultures. This regulation on non-infected cells was not perceptible in the long-term experiment with *P. salmonis *or in the assays with RTS11. Schmitten and Zakrajsek [30] demonstrated that the addition of serum to serum-starved human fibroblast cultures increased the mRNA amount for *GAPDH*. Although in our experiments, cells were maintained in a low serum concentration (5% fetal bovine serum), the addition of fresh media prior to infection could stimulate slightly the expression of *GAPDH *in the first few hours. Therefore, an important point to consider during the selection of a reference gene is whether expression could be eventually regulated by other factors than the challenge self. This differential regulation in the reference gene could lead to over- or sub-estimations of target gene expression. As well, *TUBA *showed increased expression over 48 h in both control and IPNV-infected CHSE-214 cultures, while in infected RTS11 cultures, *TUBA *was downregulated. Then, *TUBA *gene expression was influenced by IPNV infection but the direction of the influence depended on the cell type.

From our experiments using CHSE-214 and RTS11 as *in vitro *infection models for the study of immune host responses to the facultative bacteria *P. salmonis *and to the aquabirnavirus IPNV, we recommend the use of the following housekeeping genes: *UBQ*, *EF1A *and *ACTB*, as they showed an overall stable performance in both cell types under the infection conditions.

## Abbreviations

MOI: multiplicity of infection; CPE: cytopathic effect; PFU: Plaque forming unit; MEM: minimal essential medium; FBS: fetal bovine serum; RT-PCR: reverse transcription - polymerase chain reaction.

## Competing interests

The authors declare that they have no competing interests.

## Authors' contributions

AAP was the first author of the manuscript, was responsible for primer design, determined the study design and performed all experimental procedures and analyses. NCB revised critically the manuscript for important intellectual content and SHM supervised the study and revised critically the manuscript. All authors read and approved the final manuscript.

## Supplementary Material

Additional file 1 **Details of the primer pairs used for real-time PCR**. The table contains information about primer sequences, product sizes, annealing temperatures (Ta), reaction efficiencies (E), Pearson's coefficients of determination (R^2^) and melting temperatures of the amplicon (Tm) for each candidate reference gene in each cell line. Click here for file

## References

[B1] Bustin SA Quantification of mRNA using real-time reverse transcription PCR (RT-PCR): trends and problems J Mol Endocrinol 2002 29 23 39 10.1677/jme.0.0290023 12200227

[B2] Huggett J Dheda K Bustin S Zumla A Real-time RT-PCR normalisation; strategies and considerations Genes Immun 2005 6 279 284 10.1038/sj.gene.6364190 15815687

[B3] Vandesompele J De Preter K Pattyn F Poppe B Van Roy N De Paepe A Speleman F Accurate normalization of real-time quantitative RT-PCR data by geometric averaging of multiple internal control genes Genome Biol 2002 3 research0034 10.1186/gb-2002-3-7-research0034 12184808 PMC126239

[B4] Pfaffl MW Tichopad A Prgomet C Neuvians TP Determination of stable housekeeping genes, differentially regulated target genes and sample integrity: BestKeeper - Excel-based tool using pair-wise correlations Biotechnol lett 2004 26 509 15 10.1023/B:BILE.0000019559.84305.47 15127793

[B5] Andersen CL Jensen JL Orntoft TF Normalization of real-time quantitative reverse transcription-PCR data: a model-based variance estimation approach to identify genes suited for normalization, applied to bladder and colon cancer data sets Cancer Res 2004 64 5245 5250 10.1158/0008-5472.CAN-04-0496 15289330

[B6] Dheda K Huggett JF Chang JS Kim LU Bustin SA Johnson MA Rook GA Zumla A The implications of using an inappropriate reference gene for real-time reverse transcription PCR data normalization Anal Biochem 2005 344 141 143 10.1016/j.ab.2005.05.022 16054107

[B7] De Witte-Orr S Bols N Cytophatic effects of chum salmon reovirus to salmonid epithelial, fibroblast and macrophage cell lines Virus Res 2007 126 159 171 10.1016/j.virusres.2007.02.012 17391795

[B8] Tafalla C Sanchez E Lorenzen N De Witte-Orr S Bols N Effects of viral hemorrhagic septicemia virus (VHSV) on the rainbow trout (*Oncorhynchus mykiss*) monocyte cell line RTS11 Mol Immunol 2008 45 1439 1448 10.1016/j.molimm.2007.08.015 17928055

[B9] Fryer JL Lannan CN Garcés LH Larenas JJ Smith PA Isolation of a Rickettsiales-like organism from diseased coho salmon (Oncorhynchus) in Chile Fish Pathol 1990 25 107 114

[B10] Rojas V Galanti N Bols NC Marshall SH Productive infection fo Piscirickettsia salmonis in macrophages and monocyte-like cells from rainbow trout, a possible survival strategy J Cell Biochem 2009 108 631 637 10.1002/jcb.22295 19681041

[B11] Jorgensen SM Kleveland EJ Grimholt U Gjoen T Validation of reference genes for real-time polymerase chain reaction studies in Atlantic salmon Mar Biotechnol 2006 8 398 408 10.1007/s10126-005-5164-4 16676145

[B12] Ingerslev HC Pettersen EF Jakobsen RA Petersen CB Wergeland HI Expression profiling and validation of reference gene candidates in immune relevant tissues and cells from Atlantic salmon (*Salmo salar *L.) Mol Immunol 2006 43 1194 1201 10.1016/j.molimm.2005.07.009 16139890

[B13] Olsvik PA Lie KK Jordal AE Nilsen TO Hordvik I Evaluation of potential reference genes in real-time RT-PCR studies of Atlantic salmon BMC Mol Biol 2005 6 21 10.1186/1471-2199-6-21 16293192 PMC1314898

[B14] Hibbeler S Scharsack JP Becker S Housekeeping genes for quantitative expression studies in the three-spined stickleback *Gasterosteus aculeatus* BMC Mol Biol 2008 9 18 10.1186/1471-2199-9-18 18230138 PMC2254436

[B15] McCurley AT Callard GV Characterization of housekeeping genes in zebrafish: male-female differences and effects of tissue type, developmental stage and chemical treatment BMC Mol Biol 2008 9 102 10.1186/1471-2199-9-102 19014500 PMC2588455

[B16] Ganassin R Bols N Development of a monocyte/macrophage-like cell line, RTS11, from rainbow trout spleen Fish Shellfish Immunol 1998 8 457 476 10.1006/fsim.1998.0153

[B17] De Witte-Orr S Zorzitto J Sutton L Bols N Preferential induction of apoptosis in the rainbow trout macrophage cell line, RTS11, by actinomycin D, cycloheximide and double stranded RNA Fish Shellfish Immunol 2005 18 279 295 10.1016/j.fsi.2004.08.001 15561559

[B18] Bols NC Lee LE Hochachka PW, Mommsen TP Cell lines: availability, propagation and isolation Biochemistry and Molecular Biology of Fishes 1994 3 Amsterdam, Elsevier 145 159

[B19] Lannan CL Fryer JL Extracellular survival of Piscirickettsia salmonis J Fish Dis 1994 17 545 548 10.1111/j.1365-2761.1994.tb00251.x

[B20] Reed LJ Muench HA A simple method of estimating fifty percent end points Am J Hyg 1938 27 493 497

[B21] Jashes M Gonzalez M Lopez-Lastra M De Clercq E Sandino AM Inhibitors of infectious pancreatic necrosis virus (IPNV) replication Antiviral Res 1996 29 309 312 10.1016/0166-3542(96)80226-9 8739609

[B22] Marshall SH Heath S Henríquez V Orrego C Minimally invasive detection of Piscirickettsia salmonis in cultivated salmonids via the PCR Appl Environ Microbiol 1998 64 3066 3069 9687475 10.1128/aem.64.8.3066-3069.1998 PMC106817

[B23] Kibbe WA OligoCalc: an online oligonucleotide properties calculator Nucleic Acids Res 2007 35 W43 W46 http://www.basic.northwestern.edu/biotools/oligocalc.html doi:10.1093/nar/gkm234 10.1093/nar/gkm234 17452344 PMC1933198

[B24] Hellemans J Mortier G De Paepe A Speleman F Vandesompele J qBase relative quantification framework and software for management and automated analysis of real-time quantitative PCR data Genome Biol 2007 8 R19 10.1186/gb-2007-8-2-r19 17291332 PMC1852402

[B25] Watson S Mercier S Bye C Wilkinson J Cunningham AL Harman AN Determination of suitable housekeeping genes for normalisation of quantitative real-time PCR analysis of cells infected with human immunodeficiency virus and herpes viruses Virol J 2007 4 130 10.1186/1743-422X-4-130 18053162 PMC2216015

[B26] Radonic A Thulke S Bae HG Müller MA Siegert W Nietsche A Reference gene selection for quantitative real-time PCR analysis in virus infected cells: SARS corona virus, Yellow fever virus, Human Herpesvirus-6, Camelpox virus and Cytomegalovirus infections Virol J 2005 2 7 10.1186/1743-422X-2-7 15705200 PMC549079

[B27] De Boever S Vangestel C De Backer P Croubels S Sys SU Identification and validation of housekeeping genes as internal control for gene expression in an intravenous LPS inflammation model in chickens Vet Immunol Immunopathol 2008 122 312 317 10.1016/j.vetimm.2007.12.002 18272235

[B28] Fernandes JM Mommens M Hagen O Babiak I Solberg C Selection of suitable reference genes for real-time PCR studies of Atlantic halibut development Comp Biochem Physiol B Biochem Mol Biol 2008 150 23 32 10.1016/j.cbpb.2008.01.003 18302990

[B29] Sirover MA New insights into an old protein: the functional diversity of mammalian glyceraldehyd-3-phosphate dehydrogenase Biochem Biophys Acta 1999 1432 159 184 10407139 10.1016/s0167-4838(99)00119-3

[B30] Schmittgen TD Zabrajsek BA Effect of experimental treatment on housekeeping gene expression: validation by real-time, quantitative RT-PCR J Biochem Biophys Methods 2000 46 69 81 10.1016/S0165-022X(00)00129-9 11086195

