# Supplementary material for: An evaluation of potential reference genes for stability of expression in two salmonid cell lines after infection with either Piscirickettsia salmonis or IPNV
Source: BMC Res Notes. 2010 Apr 14;3:101. doi: 10.1186/1756-0500-3-101 (PMC2873344; doi:10.1186/1756-0500-3-101)
Supplement: Additional file 1 — Details of the primer pairs used for real-time PCR. The table contains information about primer sequences, product sizes, annealing temperatures (Ta), reaction efficiencies (E), Pearson's coefficients of determination (R2) and melting temperatures of the amplicon (Tm) for each candidate reference gene in each cell line. [file 1756-0500-3-101-S1.DOC]

**Details of the primer pairs used for real-time PCR**

|  | | | | | **CHSE-214** | | | **RTS11** | | |
| --- | --- | --- | --- | --- | --- | --- | --- | --- | --- | --- |
| **Target gene** | **Sense primer**  **(5´-3´)** | **Antisense primer (5´-3´)** | **Product (bp)** | **T_a_ (°C)** | **E** | **R^2^** | **Tm (°C)** | **E** | **R^2^** | **Tm (°C)** |
| *ACTB* | tatccacgagaccacctaca | atccagacggagtatttacg | 204 | 56.0 | 1.87 | 0.9983 | 83.9 | 1.91 | 0.9910 | 83.7 |
| *EF1A* | gtctacaaaatcggcggtat | cttgacggacacgttcttga | 198 | 56.0 | 1.93 | 0.9994 | 84.0 | 1.96 | 0.9989 | 84.6 |
| *GAPDH* | cctgcagtacatggtttaca | tcaatgctgaagaagactcc | 201 | 56.0 | 1.93 | 0.9993 | 83.0 | 1.93 | 0.9956 | 82.4 |
| *UBQ* | ggaaaaccatcacccttgag | ataatgcctccacgaagacg | 205 | 56.0 | 1.90 | 0.9998 | 81.8 | 1.94 | 0.9974 | 81.8 |
| *TUBA* | tactaacctcaacaggctcat | agcaagcgttggtgatctc | 215 | 56.0 | 1.88 | 0.9977 | 83.6 | 1.89 | 0.9993 | 84.0 |

For each candidate reference gene in each cell line, primer sequences, product sizes, annealing temperatures (Ta), reaction efficiencies (E), Pearson’s coefficients of determination (R^2^) and melting temperatures of the amplicon (Tm) are indicated.
